# Women’s environmental quality of life is key to their overall quality of life and health: Global evidence from the WHOQOL-100

**DOI:** 10.1371/journal.pone.0310445

**Published:** 2024-10-02

**Authors:** Suzanne M. Skevington, Kara Schick-Makaroff, Christine Rowland, Anita Molzahn, and the WHOQOL Group

**Affiliations:** 1 Division of Psychology and Mental Health, Manchester Centre for Health Psychology, University of Manchester, Manchester, United Kingdom; 2 Faculty of Nursing, College of Health Sciences, University of Alberta, Edmonton, Alberta, Canada; Universitas Padjadjaran, INDONESIA

## Abstract

Gender inequalities in health-related quality of life (QoL) are generally few and small, even in large surveys. Many generic measures limit assessment to QoL overall and its physical and psychological dimensions, while overlooking internationally important environmental, social, and spiritual QoL domains. Unique cross-cultural legacy data was collected using four WHOQOL-100 surveys of adults living in 43 cultures world-wide (17,608 adults; ages 15–101). It was first used to examined gender profiles of its five QoL international domains, and their component facets. Few significant gender differences (p < .001) were found. Women reported higher spiritual QoL than men on faith, and spiritual connection facets specifically. Men reported higher physical and psychological QoL domains than women. We aimed to identify those QoL dimensions that contribute to women’s overall QoL in health, as this information could inform gender inequalities interventions in health. Environmental QoL explained a substantial 46% of women’s overall QoL and health (n = 5,017; 17 cultures) (stepwise multiple regression adjusted for age, education, and marital status covariates). Five environmental QoL facets contributed significantly to this result; home environment offered most explanation. Age band analysis was conducted to understand when interventions might be best timed in the lifespan to improve women’s QoL. Younger women (< 45 years) reported the poorest QoL across the lifetime, and on every domain. After 45, all domains except physical QoL increased to very good at about 60, and high levels were sustained beyond 75, especially environmental QoL. Global findings show that assessing environmental, social, and spiritual QoL domains are key to fully understanding women’s QoL and health. These assessments should be prioritized in surveys that aim to improve international conservation, and public health policies.

## 1. Introduction

Gender inequalities are important as lower inequality contributes to greater subjective wellbeing [1]. Gender analysis of inequalities is widely accepted by intergovernmental organizations (IGOs) as the first and most critical step towards gender-responsive planning and programming. This is because it provides sound evidence essential to justifying policies [2], like enhancing QoL.

Health research shows that symptoms and disease are differentially perceived by women and men, resulting in distinctive styles of reporting their health during health care [3]. Reporting pain provides a suitable example [4]. Gender affects the ways professionals prescribe and treat, with consequent effects for health and wellbeing during life. Classic studies of women with chest, back or head pain [5, 6] show they were given counselling or prescriptions more often than men, whereas men were offered more injections and surgery. Recently the Rockefeller Foundation [7] has stated “*there is no such thing as gender-neutral health care”*.

Gender inequalities affect how research is conducted, health policy formed, and services implemented. Although gender differences can be interpreted as fundamental to biology, in the present study, gender is viewed as a fluid, subjective concept that is inferred by society, regardless of that person’s biological sex at birth. How people present themselves is as important to the care they receive, as their underlying physiology [8].

Biases in health research have been observed. Gender ‘blindness’ has hindered attempts to explain causal relations [9, 10]. Where gender is viewed as a ‘nuisance variable’ that introduces ‘noise’ into results, it has been deleted, controlled, or ignored, rather than investigated. The social, economic, and cultural processes supporting gender differences depend on many factors, such as the historical period of data collection, its cultural context, political system, stage of economic development; also which measures of material wellbeing, social position, health and subjective wellbeing, are selected [9]. Such issues relate to the material circumstances of poverty and work, and ‘objective’ health measures like morbidity rate [11]. The present study addresses the subjective experience.

Less is known about health and wellbeing in women than men. Some of the largest clinical trials ever conducted on major causes of mortality, for example with cardiovascular disease, exclusively recruited men (see [12]). Furthermore, it is erroneously assumed that these male findings directly apply to women. Women too can be seriously affected by these life-threatening conditions, but it is now known that they tend to report symptoms in different styles [13]. In addition, positive wellbeing is linked to biological correlates that are gender distinctive [14]. For instance, under-diagnosis of myocardial infarction in women occurs because it is detected by different biomarkers in men [15]. Serious knowledge gaps about women’s health have not been fully rectified by large scale replications. Inclusive research and unbiased policies are key to delivering appropriate clinical care for all.

Quality of life is profoundly affected by health, and vice versa. More importantly, positive wellbeing is directly related to the mortality rate, and a greater will to live [16]. Preserving positive QoL in older adults is known to benefit the physical functioning of cardiovascular and immune systems [17]. However, the best way to capture the richness of a life in order to improve it, is still debatable. Where gender differences are recorded they tend to show that men report better overall QoL than women, and this is not exclusive to high income countries [18]. As the new multidimensional measures of health-related quality of life (HRQoL) have been developed, gender differences in the dimensions of physical, functional and psychological QoL (e.g. in the EQ 5-D [19]; SF-36 [20] measures), have commonly been assessed, and generally affirm better physical and psychological QoL in men.

However, recent advances in cross-cultural methodology have enabled distinctive, important, international QoL dimensions to be newly identified, that are now included in the WHOQOL suite of instruments [21]. About the same time, a new methodology for developing measures emerged that actively involved the types of people who would eventually complete the measure and engaged them in co-designing scales. These patient- or person-reported outcome measures (PROMs) represent a new generation of subjective measures. Consequently, PROMS assessments such as the WHOQOL are more comprehensive conceptually, and more accessible to users, than earlier measures. More importantly PROMs demonstrate better psychometric properties of validity and reliability [22] than before. Opening up the development process facilitated the discovery of new QoL dimensions that were proposed and endorsed by users as both relevant and important to their QoL and health. These had been largely overlooked or undervalued in previous research, where contents were often proposed by expert scientists or clinicians.

Studies of gender differences in the related concept of subjective wellbeing (SWB) also show inconsistent results. This may because the core components of SWB, namely positive mood, negative mood, and life satisfaction [23], represent a subset of the dimensions found within the more elaborated QoL construct [24]. Furthermore, large surveys indicate that gender [25] and age [26] may only be indirectly related to SWB. National differences have been found to offset gender differences in studies examining life satisfaction like the World Values Survey, where slightly higher life satisfaction for men was recorded across three waves [27]. In the MIDUS study of mid-life, more positive social relations were found in women than men. However, this research showed more gender similarities than differences [28]. Prompted by these equivocal findings, the present study aimed to investigate QoL and health in gender groups, and its relation to age. Equivocal findings have been attributed to the minimal information obtained from one single item, often used to assesses a univariate concept like happiness or life satisfaction [29]. Four items are considered to be the minimum number needed to produce a reliable score in psychometric practice. Extraordinary events also influence outcomes, for instance, during the Covid-19 pandemic in UK, women reported less life satisfaction and happiness than men, but pre-pandemic levels were restored the following year [30].

Using one measures of multiple dimensions offers a detailed profile of different aspects of QoL and wellbeing within one single instrument. As all dimensions are measured on the same metric, comparisons between them are more accurate and legitimate than those obtained from a battery of instruments. The multidimensional WHOQOL-Bref QoL survey was administered in 23 countries, and the results showed that women reported higher social QoL, and lower psychological QoL than men [31]. The 25 facets of QoL are each evaluated by one item in the WHOQOL-Bref, so information about each dimension is limited, and less reliable than from four items that together assess each of the same 25 facets in the WHOQOL-100. The unique cross-cultural WHOQOL-100 dataset analyzed in the present study therefore enables a more robust multidimensional investigation of QoL in gender and age. The domains and facets of the WHOQOL are endorsed as important [32–34]. Gender was significant in an older adults survey of 22 cultures using the WHOQOL-Old importance measure [35], but variation in QoL and health has not been previously investigated across the entire adult lifespan for women, with this cross-cultural measure, and this is an aim of the present study.

A further aim was to investigate which WHOQOL-100 dimensions best predict women’s overall QoL and health. Here our investigation focusses on the relatively new domains of environmental, social, and spiritual QoL, where cross-cultural data on women is rarely available or analyzed. Studying environmental QoL is timely, in view of current global concerns about climate change. Environmental QoL scores from the WHOQOL-Bref are sensitive to environmental impacts [36], as a study of high visual annoyance about wind turbines shows [37]. In a review of gender differences in environmental beliefs about climate change, women were found to be more aware of risks to their QoL and health than men [38].

The present study examined cross-cultural WHOQOL-100 legacy data merged from four surveys. The first aim was to re-evaluate gender differences in subjective QoL across the multidimensional profile, to reexamine which dimensions distinguish women from men. Based on reports from previous research it was expected that women would report better QoL in the social domain, and men better QoL on psychological and physical domains [28]. Second, focusing on women, we aimed to investigate which domains and facets best predict overall QoL and health; social QoL was expected to be significant. Third, we aimed to examine how women’s QoL varies at different times in the lifespan, by analyzing different domains. This information could be used to deliver the timely implementation of interventions, to enhance women’s QoL during the lifecycle.

The WHO definition of subjective QoL frames the present work: “An individual’s perception of their position in life, in the context of the culture and value systems in which they live, and in relation to their goals, expectations, standards and concerns” [39 (p43)]. This socio-cultural model identifies the context and processes that construct subjective QoL for gender groups [40].

## 2. Materials and methods

### Samples

The WHOQOL-100 legacy dataset was collected in four cross-cultural surveys set up by WHOQOL Group collaborations between 1994 and 2004. The survey was cross-sectional and sampled using quotas. The data was conducted to develop and standardize a series of new international instruments by testing their psychometric properties. The WHOQOL-100 was the first core generic instrument in the suite of WHOQOL measures [32, 33, 41] and comprised 100 items. Modules of extra disease- and condition-specific items were subsequently developed and administered with the WHOQOL-100. They were designed with the purpose of completing the concept of QoL in a particular population. For the present study, WHOQOL-100 survey data from the original measure was merged with that from the three international modules. They assessed Spiritual, Religious and Personal Beliefs (SRPB) in the WHOQOL SRPB [42], older adults in the WHOQOL-Old [43], and HIV/AIDs in the WHOQOL-HIV [44]. WHOQOL-100 data was also collected by each new cultural centre that subsequently joined the collaboration, and followed the international protocol.

This human participant research was reviewed and approved twice: first, by the WHO Ethics Committee for the Division of Mental Health and Substance Abuse, World Health Organization in Geneva, who approved each international survey. (The reference is not available.) Second, local ethical permission was obtained by each participating cultural centre. Patients and other participants provided written, informed consent before participating in this study. The secondary analysis that is used in the present study was conducted on a data set that was anonymized, and did not require further ethics approval.

### Sampling and design

The WHOQOL-100 was designed to be used by the whole adult population. Commensurate with guidelines for the development and standardization of measures, quota sampling was used in each survey. Participating centres in each survey targeted a minimum of 300 adults overall (240 for the WHOQOL SRPB). Subgroups of gender (50% male, female), age band (50% younger, older) and health status (50% sick, well) were recruited, applying a 2 x 2 x 2 design. Representative sampling could not be applied as some countries did not have the national statistics available that could have enabled all centres to fulfil protocol requirements, had an international representative sampling frame been applied.

Every collaborating centre targeted 50% women and 50% men. For the WHOQOL-100 survey, 50% were younger (< 45 years) and older (45+ years). Adults were eligible if they were under 60 years and above the lowest adult age for that culture; this was culturally defined by every centre. The WHOQOL-Old collected three age bands of 33%: 60–69, 70–79 and 80+ years. No adult age limits were set for the WHOQOL SRPB.

Health status quotas were 80% sick and 20% well for the WHOQOL-100 survey. As this generic instrument was designed primarily for health care use, it was essential to recruit more sick participants than well and select them from the widest range of diagnostic groups. Those with serious learning disability, who were unconscious, or younger than adult, were not eligible to participate. Participants were recruited in primary care settings, hospitals, and tertiary care using convenience sampling within quotas. They were recruited in hospital wards, outpatient clinics, rehabilitation settings, primary care units, community and public health, voluntary organizations, and charities. Well people were sampled in the same locality as sick people, and explicitly included some health professionals and informal carers [32, 41] Equal health status quotas (50% sick/well) were assigned to the WHOQOL SRPB and WHOQOL-Old surveys. WHOQOL-HIV health status quotas were 25% for each of four subgroups: AIDS, HIV-symptomatic, HIV-asymptomatic, and well people. (Further details are available from publications standardizing each instrument) [42–44].

### Procedure

Secondary analysis was conducted on the merged WHOQOL-100 dataset. Survey data gathered to standardize each of the four instruments has been previously published [32, 41–44]. The WHOQOL-100 is a self-assessment measure but can be completed with the assistance of an interviewer, as required. Participants rated their quality of life “in the last two weeks” on a series of 5-point rating scales. The choice of time frame limited the influence of daily mood. The WHOQOL-100 was administered in the local language of the population, after following the WHOQOL Group international protocol for translation and cultural adaptation [45].

### Measure

The WHOQOL Group international collaboration is convened by the World Health Organization (WHO), Geneva. An advanced cross-cultural methodology was developed to improve the measurement accuracy of comparisons of QoL between diverse cultures [43]. The WHOQOL was designed for use in clinical trials, health research, and clinical practice. In the WHOQOL-100, 25 facets of QoL are clustered and scored within one of six domains reflecting its conceptual structure: physical, psychological, independence, social, environmental, and spiritual QoL. A general facet covering overall QoL and health, provides an additional overarching assessment (Table 1). Facets contain four items scored on one of several 5-point rating scales. Raw domain scores are transformed onto a scale from 0 to 100, so that domains containing unequal numbers of facets can be compared. Transformed scores around 50 indicates that QoL is okay; below 50 QoL is poor or very poor. Scoring over 60 indicates good QoL, and above 70, very good QoL.

**Table 1:** Domains and facets of the WHOQOL-100 extended by the WHOQOL SRPB.

Overall Quality of Life and General Health					
Physical	Psychological	Independence	Social	Environment	Spiritual, Religious & Personal Beliefs
Pain & discomfort	Positive feelings	Mobility	Personal relations	Physical safety & security	Meaning in Life (replaces Spirituality)
Energy & fatigue	Thinking, learning, memory & concentration	Activities of daily living	Social support	Home environment	Spiritual connection
Sleep & rest	Self-esteem	Dependence on medication/treatment	Sex life	Financial resources	Purpose in life
	Body image & attractiveness	Working capacity		Health & social care	Awe & wonder
	Negative feelings			Information & skills	Wholeness & integration
				Recreation & leisure	Spiritual strength
				Physical environment	Inner peace
				Transport	Hope & optimism
					Faith

Sociodemographic and health information was collected about gender, age (date of birth), highest educational level completed, marital status, health status and illness type. Gender was self-reported as a binary (male/female) category. No ‘other’ gender category was provided as this was not the research practice when the data were collected. In the WHOQOL-100 field trial and WHOQOL SRPB pilot survey, health was rated on a 5-point scale: 1—very poor, to 5—very good. The two other surveys collected heath in binary categories of yes/no. The WHOQOL-HIV field study asked whether they were currently ill (yes/no). The WHOQOL-Old study recorded healthy or unhealthy.

Replicating the WHOQOL-100 methodology, an international WHOQOL SRPB Group [39] enlarged the original WHOQOL-100 spiritual QoL domain consisted of one spirituality facet. Following development and modelling, the 32 items in eight additional SRPB facets were scored together with the original spirituality facet to make nine facets. To avoid ambiguity, spirituality was relabeled meaning in life which reflected its contents (Table 1). Only the WHOQOL SRPB dataset could be used to examine gender differences in 33 QoL facets.

### Analysis

Variables and cases were checked for missing data (SPSS v. 22) [46]. Cases were deleted following WHOQOL-100 manual procedures [47], where 20% or more responses were missing. Domain scores were not calculated if two or more items were omitted (one for the social domain). The WHOQOL-100 was scored as six QoL domains, and its raw domain scores transformed (0–100). As modeling of later instruments have indicated that five domains (WHOQOL-SRPB [42] and WHOQOL-Combi [48]) or four domains (WHOQOL-Bref) [32] can be appropriate, these scoring systems are adopted for some of the present analysis, as needed. For the life span analysis, age was recoded into five bands of years: 1. 15–29, young adults; 2. 30–44, early middle age; 3. 45–59, late middle age; 4. 60–74, early old age; 5. 75+ years, late old age.

Tests for normality, multicollinearity, and homoscedasticity were conducted on each survey, then on the merged dataset. Departures from normality were largely acceptable and outliers low (< 20).

To examine gender differences and similarities in six domains and all facets, one-way ANOVA was conducted on the merged sample. The Welch test (W) replaced the F-test statistic, as we could not assume equal variances. Adjusting for multiple post-hoc testing (Scheffe test), a significance level of p < .01 was applied. As data on all nine spiritual facets was only collected by the WHOQOL SRPB survey, the analyzed sample was smaller.

Multivariate analysis was used to examine which QoL dimensions best predict overall QoL and health (dependent variable) for women. Stepwise multiple regression models were preceded by the pre-selection of socio-demographic and health status variables, so that the best covariates were entered into the model. Their Pearson correlations with other key variables were first calculated; r = .3 to .7 was considered acceptable. The remaining sociodemographic and health variables that entered as significant into the first step of the model. were then confirmed as model covariates. Two multiple regressions were conducted; the first assessed the six QoL domains as independent variables. In the second, the 24 specific facets of the WHOQOL-100 were substituted. The entry and removal criterion at each step was p < .05. Other indices evaluated included plots of Z-residuals, the Durbin-Watson statistic, and Scheffe test.

A multivariate analysis of covariance (MANCOVA) (General Linear Model; GLM) with repeated measures for six domains was used to examine the effect of age in five bands on women’s QoL. Covariates of educational level and marital status adjusted the results; alpha was p < .05. Pillais’ overall multivariate test was conducted; F-tests and eta squared was calculated for main effects and interactions. Multiple paired comparison tests between pairs of age bands within each domain were adjusted by the Bonferroni test. Estimated marginal means for domains adjusted by the covariates, were plotted against age.

## 3. Results

### Sample description

WHOQOL-100 data was collected in 43 cultural centres located in 35 countries world-wide and drawn from all inhabited world regions. There were 17,608 uncleaned cases: 9,080 women, and 8,246 men. The mean time taken to complete 100 items (or 132 for the WHOQOL-SRPB) was around 20 minutes. The ‘About You’ section of the questionnaire was situated on the last page, so sociodemographic and health questions were sometimes missed. Fatigue affected completion rates and caused missing data. Other factors included the burden of illness, busy clinics, and a shortage of personal time. Reasons for refusal included lack of privacy, and limited literacy in some countries.

Following screening and cleaning, multivariate analysis was conducted on the complete data from 11,241 adults in 17 countries: 5,017 women and 6,224 men. Of these, 50.25% reported they were sick and 49.74% were well. Eight additional spiritual facets in the WHOQOL SRPB were completed by 3,479 participants.

Contributions from the centres and their samples, were as follows: Bath 934 and Edinburgh 303, U.K.; Leipzig 433, Germany; Tilburg 413, The Netherlands; Barcelona 847, Spain; Paris 456, France; Geneva 161, Switzerland; Rome 376 and Naples 151, Italy; Prague 350, Czechoslovakia; Budapest 304, Hungary; Zagreb 300, Croatia; St Petersburg 300, Russia; Dniepropetrovsk 300, Ukraine; Copenhagen 467, Denmark; Oslo 372, Norway; Umea 315, Sweden; Vilnius 927, Lithuania; Alexandria 240, Egypt; Harare 450, Zimbabwe; Eldoret 480, Kenya; Beer Sheva 928, Israel; Izmir 574, Turkey; Melbourne 670, and Victoria 343, Australia; Madras 412, New Delhi 499 and Bangalore 592, India; Tokyo 937, Japan; Bangkok 728, Thailand; Guangzhou, 737 and Hong Kong 319, China; Phnom Penh 33, Cambodia; Kubang Kerian 240, Malaysia; Panama City 300, Panama; La Plata 225, Argentina; Porto Alegre 1044 and Santa Maria 253, Brazil; Montevideo 256 and Calabria 251, Uruguay; Edmonton and Victoria 430, Canada; Seattle 535, U.S.A. Sample sizes ranged from 33 to 937. Where centres had collaborated on more than one instrument, multiple samples are included.

The grand mean shows that globally, QoL is good for women (mean 61.27; SE .18), and for men (mean 62.66; SE .19). Together the international samples collected data from across the entire adult lifespan, from 15 to 101 years. The five age bands contained fairly equal gender groups. The proportion of women in each age band was: 15–29 13%; 30–44 14%; 45–59 10%; 60–74 41%, and 75+ 22%, with 60–74 most numerous.

Recoding some variables was needed to adjust for small numbers in some adjacent categories. Marital status proportions in women were 12% single, 50% married, 4% living as married/partnered, 9%, separated/divorced, and 25% widowed; partners and married were merged. Self-reported health status for women was reported as 3,168 sick and 3,265 well. Women’s highest educational level was: .3% no education, 25% primary education, 29% secondary education, 35% college/university, and 4% postgraduate; the two lowest categories were merged. Educational level is an acceptable indicator of social class [49].

In total, 30.3% of health status data was missing. This was a problem for the WHOQOL-Old survey as data was not available from some centres. Where this occurred, cases were necessarily excluded, as the variables pertinent to the research question could not be analyzed. An additional problem was that depending on the survey, health status was recorded in binary form or on a 5-point interval rating scale, so the scores could not be satisfactorily combined for analysis. Consequently, samples of data resulting from these different methods groups were halved, and each had a different distribution of cultures. If analyzed, such results could mislead conclusions about gender.

### Gender differences in quality of life

Domain scores of the total WHOQOL-100 dataset (n = 17,608) were analyzed for gender and two of the six domains showed significant gender differences (W) (p < .01) (Table 2). Men reported better physical and psychological QoL than women, confirming some previous research. Domains on independence, social relations, environment and spiritual QoL did not show significant gender differences. The finding that social relations QoL domain was similar for women and men, did not support the hypothesis that there would be a difference.

**Table 2:** Gender differences in six quality of life domains in the WHOQOL-100. (women n = 9,080; men n = 8,246) (# a single facet domain).

WHOQOL-100 Domains	Women Mean (SD)	Men Mean (SD)	Total Mean (SD)	W	p	Cohen’s d
Physical	57.82 (14.88)	58.69 (14.62)	58.24 (14.76)	17.95	.0001*	-0.06
Psychological	61.22 (12.48)	61.77 (12.59)	61.48 (12.54)	12.85	.0001*	-0.04
Independence	61.91 (17.16)	61.90 (16.46)	61.91 (16.83)	.003	.953	0.00
Social relations	64.59 (15.85)	63.26 (16.20)	63.96 (16.03)	.003	.956	0.08
Environment	63.28 (14.95)	62.18 (15.15)	62.76 (15.06)	1.966	.116	0.07
Spiritual #	62.46 (22.59)	59.99 (22.69)	61.29 (22.67)	3.199	.074	0.11

Among multiple facet tests, there were many more gender similarities in QoL than differences (see Tables 2 and 3). Of the 33 facets, only six showed significant gender differences at p < .01. Although the spiritual domain showed no overall difference, women reported significantly higher QoL than men on two of its facets, namely spiritual connection with a being or force, and faith.

**Table 3:** Gender differences in 33 quality of life facets in the WHOQOL-100 (n = 17,339) and WHOQOL SRPB (*italics*) (n = 3,714).

Facets	Women Mean (SD)	Men Mean (SD)	W	P	Cohen’s d
General QoL & Health	14.07 (3.19)	13.94 (3.31)	1.584	.209	0.04
Pain & discomfort	11.76 (3.79)	12.03 (3.87)	.011	.918	-0.07
Energy & fatigue	13.24 (3.42)	13.56 (3.39)	11.701	.001*	-0.09
Sleep & rest	14.27 (3.89)	14.63 (3.83)	18.050	.0001*	-0.09
Positive feelings	13.29 (3.03)	13.24 (3.11)	1.839	.175	0.02
Cognitions	13.93 (2.71)	14.09 (2.75)	.495	.482	-0.06
Self-esteem	13.92 (2.86)	14.19 (2.95)	24.574	.0001*	-0.09
Body image & appearance	14.99 (3.27)	15.59 (3.20)	52.795	.0001*	-0.19
Negative feelings	11.16 (4.19)	11.69 (4.40)	3.317	.069	-0.12
Mobility	14.94 (3.93)	15.07 (3.88)	.035	.852	-0.03
Activities of daily living	14.89 (3.58)	14.91 (3.53)	1.553	.213	-0.01
Dependence on medication/treatment	12.38 (5.15)	12.53 (5.10)	1.329	.249	-0.03
Working capacity	14.17 (3.92)	14.18 (3.84)	.192	.661	0.00
Personal relations	15.23 (2.81)	15.17 (2.86)	2.508	.114	0.02
Social support	14.41 (3.29)	14.08 (3.26)	.143	.706	0.10
Sex-life	13.16 (3.40)	13.05 (3.59)	.705	.401	0.03
Physical safety & security	13.97 (2.97)	13.92 (2.99)	5.760	.017	0.02
Home environment	15.12 (3.26)	14.78 (3.29)	.007	.935	0.10
Financial resources	13.46 (4.12)	13.09 (4.10)	.021	.885	0.09
Health & social care	13.69 (2.91)	13.52 (2.98)	.062	.804	0.06
Information & skills	14.06 (3.04)	13.91 (3.08)	1.542	.215	0.05
Leisure & recreation	13.59 (3.34)	13.42 (3.33)	1.826	.177	0.05
Physical environment	14.02 (2.97)	13.92 (2.93)	.290	.590	0.03
Transport	15.04 (3.68)	15.01 (3.64)	6.061	.014	0.01
Spiritual, religious & personal beliefs or Meaning in Life	13.99 (3.61)	13.59 (3.63)	3.199	.074	0.11
Spiritual connection	12.66 (4.40)	12.13 (4.50)	13.051	.0001*	0.12
Purpose in life	14.39 (2.97)	14.33 (2.92)	.372	.542	0.02
Awe & wonder	14.09 (3.30)	13.92 (3.24)	2.445	.118	0.05
Wholeness & integration	13.77 (2.97)	13.77 (2.98)	.005	.945	0.00
Spiritual strength	13.63 (3.41)	13.49 (3.34)	1.438	.231	0.04
Peace & harmony	13.14 (3.08)	13.38 (2.96)	5.556	.018	-0.08
Hope & optimism	13.88 (3.10)	14.08 (2.99)	4.024	.045	-0.07
Faith	13.79 (4.35)	13.35 (4.41)	9.534	.002*	0.10

Key

* p < .01

Men had higher QoL on the four facets of energy and sleep in the physical domain respectively, and self-esteem and body image in the psychological domain, confirming predictions that QoL in these domains would be higher for men. However, the two facet differences found on self-esteem and body image are not widely reported in the literature.

### Which dimensions of QoL best predict the overall health -related quality of life of women?

Following Pearson correlations, and the selection of covariates for the models, age, marital status, and educational level were entered together, at step 1 of each model. All six domains made a significant contribution to the final model as shown by F-change (p = .0001) at each step (Table 4). Together these domains explained 67.6% of variance in overall QoL and health (Durbin-Watson = 1.81). R square adjusted (R sq. adj.) for the covariates of age (beta β = -.027) and marital status (β = -.020) accounted for 7.8% of the variance in the final model, but educational level was not significant.

**Table 4:** Multiple regression modelling to show the WHOQOL-100 facets that significantly predict women’s overall QoL and health.

Model of Facets	Adjusted R Square	Std. Error	R Sq. Adj. Change	F-Change	Sig. F- Change	β
1 Covariates	.081	2.9773	.081	144.03	.0001	
2 Positive feelings	.512	2.1698	.431	4304.26	.0001	.188
3 Activities of daily living	.617	1.9209	.106	1345.04	.0001	.073
4 Personal relations	.657	1.8193	.039	560.12	.0001	.129
5 Home environment	.674	1.7719	.018	265.68	.0001	.089
6 Energy	.690	1.7304	.015	237.46	.0001	.125
7 Financial resources	.698	1.7058	.009	142.13	.0001	.107
8 Recreation & Leisure	.705	1.6869	.007	110.61	.0001	.089
9 Self-esteem	.709	1.6749	.004	70.90	.0001	.077
10 Mobility	.712	1.6653	.003	57.63	.0001	.065
11 Health & social care	.715	1.6565	.003	52.64	.0001	.058
12 Sex-life	.717	1.6518	.002	28.84	.0001	.047
13 Sleep	.718	1.6483	.001	21.95	.0001	.042
14 Social support	.719	1.6453	.001	18.35	.0001	.040
15 Dependence on medication/treatment	.720	1.6430	.001	14.65	.0001	-032
16 Working capacity	.721	1.6408	.001	14.05	.0001	.043
17 Spirituality	.721	1.6399	.0001	6.14	.013	.022
18 Physical environment	.721	1.6391	.0001	5.87	.015	.024

Footnote: Durbin-Watson = 1.88 (df = 1) n = 4873–4857. Covariates: marital status, educational level, age

Environmental QoL was the first domain to be entered into the model at step 2, and accounted for a substantial 46.2% of the variance (β = .336). Psychological QoL (7.6%) (β = .182) entered at the third step. The social relations domain explained a further 2.9% but showed a larger beta than the psychological domain (β = .222), so although small, the contribution confirmed that social relations are a significant predictor of women’s overall QoL and health. Successive models introduced the independence (2.6%) (β = .163) domain, physical domain (.3%) (β = .085), and lastly spiritual domain (.2%) (β = .054).

These results demonstrate that every international domain in the WHOQOL-100 makes a significant contribution to women’s QoL and health globally.

The second regression assessed the contributions of 24 core QoL facets to overall QoL and health. The same covariates accounted for 8.1% of the variance. Table 4 shows that 72.0% (R sq. adj.) of overall QoL and health is explained by 17 facets, with the first five accounting for the most variance.

Positive feelings of happiness and contentment in the psychological domain was the most influential facet, as it explains 43% of the variance. This was followed by four models (domains in brackets) that entered activities of daily living (10.6%) (independence), personal relationships 3.9% (social), home environment 1.8% (environment), and energy and fatigue 1.5% (physical). It is notable that among these, personal relationships had the second largest beta, then energy, being more important than activities. Each of these five facets are drawn from a different domain, so illustrates the wide-ranging and distinctive WHOQOL domains that comprise women’s QoL and health globally.

Subsequent models in Table 4, added 12 more facets. Although all were significant, they explained very little (< 1%) of the variance. However, it was worth noting that the two remaining social domain facets on sex-life and social support gave minor contributions to overall QoL and health, adding our support to the prediction that social relationships QoL is salient to women, as previously found.

Five of the eight environmental QoL facets were included in this facet analysis. Perceptions of the home environment was the most influential of these, although financial resources contributed a larger beta. Opportunities for recreation & leisure showed the same effect size as home environment. Access to health & social care was minor, and physical environment negligible. Nevertheless, as a cluster, these results underscore the importance of environmental QoL to women’s overall QoL and health, and its multi-facetted nature.

### How does health-related quality of life vary for women in different age groups?

**Fig 1:**
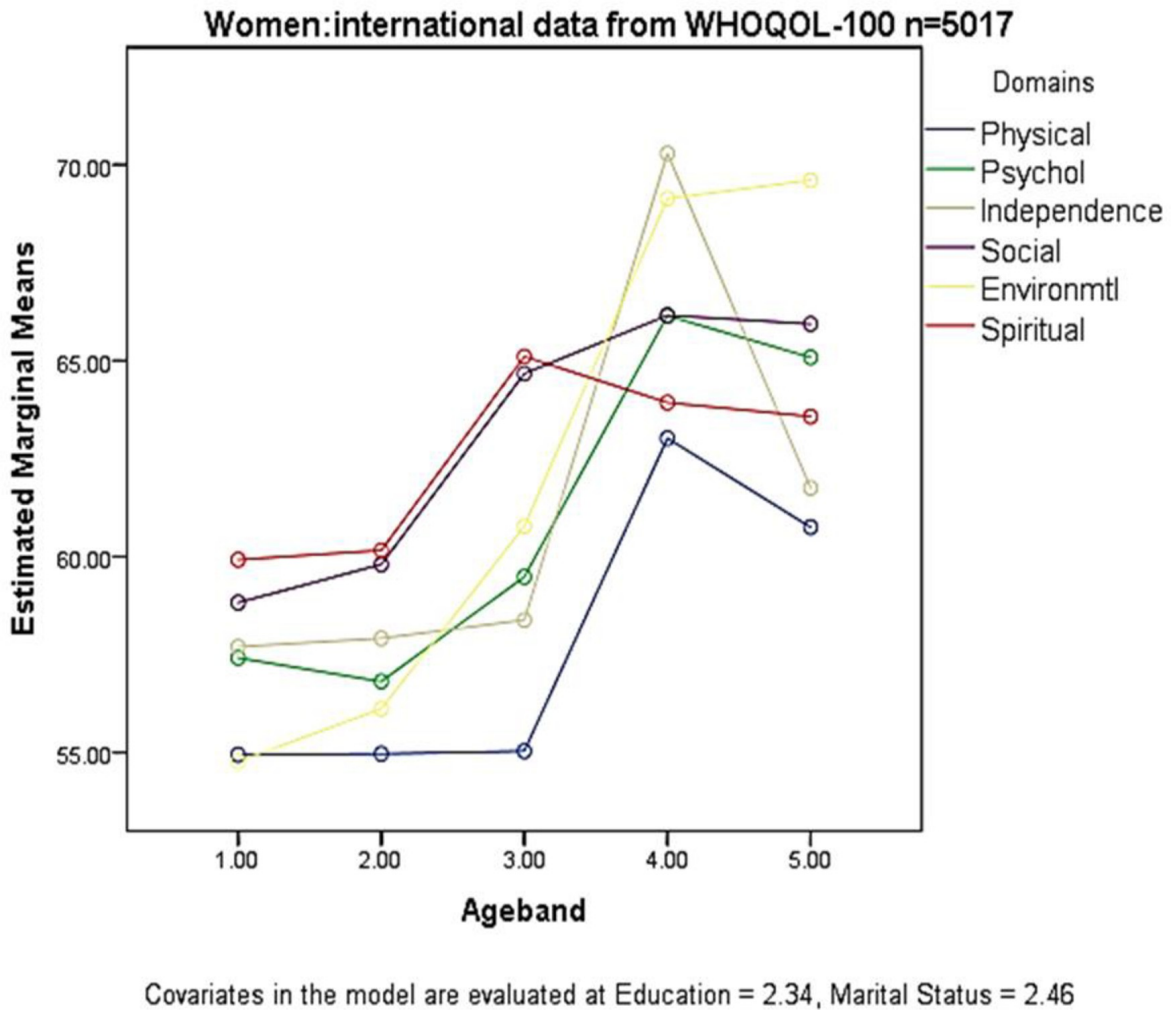
Plots of six quality of life domains and five age bands (MANCOVA).

The QoL of women (n = 5,017) was examined at different ages across the lifespan. Each variable had a significant overall multivariate effect (Pillais trace, p < .0001), but age explained the largest part of the variance (eta = .094). Table 5 shows significant improvements to women’s QoL as age increases, in the domains x age band interaction (F = 35.551; p < .0001; eta = .036). Plotted estimated marginal means for six domains against sequential age bands are illustrated in Fig 1. The results show consistent upward trends, illustrating the QoL improvements in Table 5. Covariates were significant in the model, but marital status had greater impact than educational level. Married and cohabiting women reported poorer QoL. Higher educational level was associated with better QoL, as found previously [49].

**Table 5:** 

Quality of life of women in different age bands as assessed by the WHOQOL-100 (MANCOVA)						
Source	Mean Square		F	df	P	Partial Eta Squared
Age band	98993.78		129.108	4	.0001	.094
Education	87495.62		114.112	1	.0001	.022
Marital Status	48539.07		63.305	1	.0001	.013
Error	766.75			4977		
**Results of Multivariate tests of Effects (Pillais’ Trace)**						
**Effect**	**Value**	**F**		**df**	**Sig.**	**Partial Eta Squared**
Domains of QoL	.029	30.167		5	.0001	.029
Domains * Age band	.145	37.551		20	.0001	.036
Domains * Education	.012	12.303		5	.0001	.012
Domains * Marital status	.023	22.986		5	.0001	.023

Overall, the social domain showed the highest overall QoL of all domains and was good (mean = 63.08). Environmental QoL showed the greatest change in QoL across the lifespan. It was one of the two poorest domains for young women, but increased throughout midlife to a very high level that was maintained in the two oldest age bands.

Differences in QoL between each pair of age bands within every domain, were all highly significant (p < .0001). The poorest QoL was reported by the youngest women of 15 to 29 years. Physical and environmental QoL was poorest between 15 and 44 years, although levels were acceptable. During this period, social and spiritual QoL was best, and good. Quality of life improved during late middle age (ages 45–59) becoming good (mean = 60.6), on every domain except physical QoL, which remained poorer. Physical QoL was the poorest domain (57.7) across the age range. Spiritual QoL peaked in late middle age and was good (62.5). Quality of life peaked in five domains between ages 60 to 74, and approached very good (mean = 66.4). Independence and environment domains were particularly good at this time of life. Quality of life remained good to very good (mean = 64.5) after 75 years, to the end of life; the exception was a sharp decline in independence QoL at this age. Environmental QoL was sustained at the highest level after age 60.

## 4. Discussion

It is widely assumed that men have better QoL than women, although scientific support for this belief is equivocal, and the international results of the present study indicate that this assumption is only partially correct. This survey of subjective QoL, gender and age used data from the WHOQOL-100 to assess 11,241 adults from 17 countries. As this instrument is a state-of-the-art PROMs, and a cross-cultural tool, the results have global significance and implications.

This study is timely and important in view of widespread global concerns about gender inequalities and its negative outcomes like wasted capabilities, inadequate income, and poorer family life [50]. Gender mainstreaming programs were introduced by intergovernmental organizations (IGOs) to rebalance gender inequalities, after technical strategies proved insufficient to change bias, discrimination, and inequality. These mainstreaming programs sought to address inequality through systemically changing organizational and governmental structures, and the directives were issued from the highest levels of IGOs in the health [51], education [52, 53], poverty, and work [2] fields. However, mainstreaming did not create the expected ethical imperative in relevant government ministers, who have been reluctant to designate gender responsive budgeting that could implement capacity building programs for women. It seems that gender is ‘everyone’s problem, but no one’s responsibility’ [54]. Nevertheless, gender analysis *per se*, is widely acknowledged to be the first, and most critical step towards gender-responsive planning and programming [2], as this evidence is essential to underpinning policy. The present findings on QoL demonstrate a global case, so offering a considerable statistical resource to international policy makers, supported by a suite of well-standardized cross-cultural WHOQOL tools.

Several analyses of associations between gender and subjective QoL in the present study resulted in more a nuance interpretation of the research questions than conventional gender differences alone. An advantage of the present study was being able to collect richly detailed multidimensional evidence by teasing out facets within each broad domain. Facet assessment is particularly useful for targeting specific areas of poor QoL, where change is needed. For instance, in health situations, results from WHOQOL profiles offer clinicians the necessary evidence to pragmatically select the most effective intervention for their patients. Where treatments are designed to refresh sleep or lower fatigue, this precise specific facet evidence is more useful than abstract information about poor overall QoL.

Finding more gender similarities than differences in the present study, reaffirms previous findings [28]. Of 33 QoL facets investigated, gender differences emerged on only six facets from three domains. Here facet information proved more enlightening than domain results, reflecting some previous findings. Although QoL facets on faith, and spiritual connection scored higher for women, the spiritual domain overall was no different. Women reported lower physical and psychological QoL overall than men supporting previous research, but only four facets out of 12 in these domains showed differences. Women reported poorer quality sleep and rest, and less energy with more fatigue in the physical domain, than men. Poorer body image and appearance, and lower self-esteem were key components of women’s poorer psychological QoL overall. Where QoL is poorer, this empirical evidence of inequality can support the promotion of the design and implementation of new policy at national and international levels. It can also lead adjustments to clinical practice, and steer changes in research methodology.

These findings supplement qualitative research into tiredness and exhaustion among women [55]. They reflect the costs to QoL and health of trying to satisfy the many gendered expectations of contemporary life. These outcomes appear to illustrate a struggle expressed by women to fulfil multiple roles in the family, at work, and in society. Cross-cultural results have brought to light these features which taken together, underscore tiredness as a global phenomenon for women, not only Western lifestyles.

Capacity building programs for women could address their poorer self-esteem and body image by engaging social support, and developing closer personal relationships. It is important that programs are instigated by women for women as they alone are aware of the most appropriate ways of improving their poor QoL. This proposal for action is underpinned by evidence for a theory of tending and befriending [56], which shows that females become affiliative when under stress, as they turn to others, and nurture their offspring. Age analysis in the present study indicates that schemes for younger women under 45 must become a higher priority to raise their poorer QoL. Programs should be tailored to meet the younger generation’s sociocultural QoL needs, and to do this by engaging young women in program design, implementation, evaluation, and promotion.

It was expected that women would report better social QoL than men but this was not supported. Nevertheless, several other analyses indicate that social relations, environment, and spiritual domains are relevant to women’s lives. Social QoL proved to be a significant predictor of women’s overall QoL and health in the domains regression and the second largest contributor despite the small variance. Of the three social facets in the model, personal relationships best explained women’s overall QoL and health, completed by very small additional contributions from sex-life and social support. This detail would have been missed had we only examined gender differences.

Looking at QoL at different ages, social QoL was good in early adulthood and continued to improve after 45, increasing steadily with each successive age band across the lifetime. It peaked in the retirement years, being very good between 60 and 74, and sustaining this high level into late life. These results indicate that social relationships become a source of good QoL to women, as life progresses [57]. As popular generic QoL measures like the EQ 5-D and SF-36, contain minimal social content, much less attention has focused on social QoL compared to physical and psychological domains. Until assessments that include substantial social and environment domains are widely included in research and practice, women’s QoL will not be adequately evaluated and recorded, e.g. in government statistics, while women are deprived of the means to report their QoL on issues that are central and relevant to their life.

Findings about environmental QoL showed the relevance of this QoL dimension to women internationally. A substantial 46% of overall QoL and health was explained, and this was three times greater than all the remaining domains combined, demonstrating the heavy weight given by women to environmental QoL. Also, the greatest change in QoL across age was for the environment domain, from being one of the poorest domains in young adulthood where QoL was barely acceptable at that time, increasing in midlife and reaching a peak at around 60 years. Furthermore, environmental QoL remained very good for women after 75 years.

Quality of life related to home environment was the most important facet of environmental QoL in women, as it was entered first of the five domains into the model and had the largest domain effect size. A cluster of four smaller but significant environmental facets indicates the diverse aspects of the environment that supply good QoL to women. Traditionally spending more time in the home, many women perceived the home environment as central to the quality of their life, investing in it emotionally and financially. Commensurate with tending and befriending theory, they can make it a safe, comfortable, attractive place for the whole family to be nurtured away from life’s stresses. However, our data does not answer questions about whether women are tuned into their environmental QoL because of responsibilities for health and wellbeing in their family. This needs further research across gender groups.

Quality of life relating to financial resources was entered seventh, as the second environment facet included and showed a larger effect size than home environment indicating its importance. Here our items address whether women perceived they have sufficient money to meet their needs. This topic taps into perceived standards of living and has resonance with the economics of wellbeing. The facet reflects global concerns about being able to provide sustenance, warmth, and protection, to maintain family health and wellbeing [50, 58]. Although the contribution was very small, physical environment QoL includes issues like pollution and flags up women’s wider concerns about how environmental conditions in their locality pose risks to health and QoL [38]. As the effects of global climate on biodiversity and health are becoming increasingly salient in wellbeing research [59], together these facet findings could be used to implement conservation policies [60]. Related to this, is access to recreation and leisure. Ballew et al [38] recommend that public education and outreach programs should supply environmental knowledge, which should enhance environmental QoL. Access to good quality health and social care is relevant to the work of health professionals and policy makers who are responsible for service delivery. Environmental QoL must become central to planning public health programs, and delivering health and social care. Knowing that these environmental QoL facets are of central importance to women could act as a motivator for behavior change in these directions, when policies and interventions are designed using well documented behavior change strategies [61]. As almost all other generic QoL instruments do not assess environmental QoL, or its array of environment facets available in the WHOQOL, this is an original finding, although the contributions from the cluster of the last four related facets is small.

Women derived better QoL from spiritual sources than men, predominantly from faith, and spiritual connections with a higher being or force. Previous WHOQOL SRPB studies have shown that these facets are more highly rated by religious participants, than those holding spiritual or personal beliefs [62], and could also offer sources of religious coping [63]. Detecting this detail in spiritual QoL was only possible because nine facets were available for assessment.

As spiritual QoL remains taboo in health consultations, training will be needed to orientate health professionals towards spiritual QoL as it is often overlooked in general practice. This initiative would resonate with a holistic, person-centred approach to care, that is under consideration by NHS healthcare providers in UK: https://www.cqc.org.uk/publications/themed-work/inpatient-coronavirus-experience-care Sensitive debates with stakeholders are also needed to move spiritual QoL practice into the mainstream, so the topic is normalized as legitimate, before it becomes accepted as a routine area of inquiry. This should be an aim of health services seeking to deliver comprehensive care, especially to multi-cultural societies.

Good QoL relating to mental health is important [34], but as expected, was poorer for women than men. Nevertheless, psychological QoL was a modest predictor of overall QoL and health explaining 7.6% of the variance. Within this domain, positive feelings of happiness and contentment were most important to women’s psychological life, and to their overall QoL and health globally. Although the impact of moods like happiness are well known, they are more transient than contentment which is a more stable concept that persists over a period. Consequently, QoL assessed over the last two weeks is less affected by undue influence from mood of the day when tested.

Gender differences showed that women report poorer QoL from self-esteem, and from body image and appearance. These results may be interpreted as lower self-worth, lack of self-confidence, or modesty in some cultures. Relevant to health care, these gendered QoL beliefs can influence when and from whom women are inclined to seek a health consultation when ill, and how they respond to advice and treatment; the implications are widespread.

Physical QoL was one of the poorest QoL domains for women and remained lowest until they reached 60. This result contrasts with other domains where visible QoL improvements occurred after 45. As we did not collect biological measures of ageing, reasons for delays in improvements to physical QoL remain uncertain. A spike in independence QoL during early old age (60–74) may reflect new opportunities for increased physical exercise, and less stress after retirement, when health might still be good.

There were several limitations to the present study. This cross-sectional, cross-cultural data was collected over 10 years, so causal sequences are uncertain; as historical data, cautious interpretation is needed. Data was reduced for multivariate analyses due to inaccessible sociodemographic and health information from some WHOQOL Old centres. Data was also missing due to the long length of the measures (1100 and 132 items). Also fatigue or overlooking the last page of the questionnaire where the sociodemographic and health data was collected. It was also due to refusals, item omissions (e.g. sex life) and administrative and data handling difficulties in some centres.

Nevertheless, a study strength is that sufficient complete cases were available for the final analyses, and this highly multidimensional international data provides finely tuned insights that are unique. Study strengths include the unusual breadth of cultures world-wide collected from developing and developed countries, using a common internationally agreed protocol. Many more centres are now engaged in developing a language version so West Africa and Central Asia will be better represented in future. Although the WHO definition and concept are still widely used, an international collaboration should update them and include the many new centres that have subsequently standardized a WHOQOL language version since the original work on the WHOQOL-100. Currently over 120 language versions of the WHOQOL-Bref short form [31] exist.

## 5. Conclusions

Methodology plays an important role when interpreting the QoL of gender groups. The multidimensional WHOQOL with its supplementary SRPB module, assessed a wide array of facets and domains that were not available from other international generic profiles. This enabled gender inequalities to be more systematically examined across many more dimensions than before, and in a sample containing diverse countries world-wide. Systematic international evidence on environmental, spiritual, and social QoL has been largely invisible to policy makers to date and this has been remedied. A limitation to any research, is that you can only discover what your measures allow.

In common with other large-scale surveys, the findings reaffirm that there are more gender similarities in QoL than differences. This signifies that the field should move towards conducting comprehensive systematic reviews, before collecting new data. The regression analyses offered unusual insight into the priority given to environmental QoL by women. The analysis revealed interesting new details about five dimensions of environmental QoL, that have particular impact on overall QoL and health globally.

An analysis of age showed that women’s environmental QoL increases as life progresses, and this was true with all other domains except physical QoL. Environmental actions by young adults that draw public attention to climate change and the damage to the environment, may be motivated by their poorer environmental QoL before 45 years, and fuel a desire to improve it. Very good environmental QoL in older women may provide reason for them to work towards retaining this valued feature of life, for future generations. This could be the topic of future research, as the data for the present study was collected before it was widely appreciated that the effects of climate change and biodiversity loss would depend on changing human behavior. While a strength of this research is in the use of the 4-item facet scores from the WHOQOL-100 and WHOQOL SRPB, to test the hypotheses, multilingual short-form assessments of the WHOQOL-Bref [31] and WHOQOL-Combi [48] would be suited to large scale surveys set up by intergovernmental organizations, and national governments, who are interested in gathering evidence to evaluate projects such as the Sustainable Development Goals [36, 64].

## References

[1] Audette AP, Lam S, O’Connor H, Radcliff B. (E)Quality of Life: A Cross-National Analysis of the Effect of Gender Equality on Life Satisfaction. J Happiness Stud. 2019;20(7).

[2] OECD. Innovative approaches to gender equality. Paris: OECD Development Centre, OECD, Paris; 2010.

[3] Macintyre S, Hunt K, Sweeting H. Gender differences in health: are things really as simple as they seem? Soc Sci Med. 1996 Feb;42(4):617–24. doi: 10.1016/0277-9536(95)00335-5 8643986

[4] Skevington S. M. (1995). Psychology of pain. Oxford, England, John Wiley & Sons

[5] Verbrugge LM, Steiner RP. Prescribing drugs to men and women. Health Psychol. 1985;4(1):79–98. doi: 10.1037//0278-6133.4.1.79 4018001

[6] Verbrugge LM, Steiner RP. Another look at physicians’ treatment of men and women with common complaints. Sex Roles. 1984 Dec;11(11–12):1091–109.

[7] Rockerfeller Foundation. Universal Health Coverage [Internet]. 2023. Available from: https://www.rockefellerfoundation.org/initiative/universal-health-coverage/

[8] Thompson AE, Anisimowicz Y, Miedema B, Hogg W, Wodchis WP, Aubrey-Bassler K. The influence of gender and other patient characteristics on health care-seeking behaviour: a QUALICOPC study. BMC Fam Pract. 2016 Mar 31;17:38. doi: 10.1186/s12875-016-0440-0 27036116 PMC4815064

[9] Macintyre S. Inequalities in health: is research gender blind? In: Leon DA, Walt G, editors. Poverty, Inequality, and Health. Oxford, UK: Oxford University Press; 2000. p. 283–93.

[10] Criado Perez C. Invisible Women: Exposing Data Bias in a World Designed for Men. London: Penguin Random House; 2019.

[11] Benzeval M, Judge K, Whitehead M. Unfinished Business. In: Benzeval M, Judge K, Whitehead M, editors. Tackling Inequalities in health: an agenda for action. London: Kings Fund; 1995. p. 122–40.

[12] Hemmingway H. Social and psychosocial influences on sudden cardiac death, ventricular arrhythmia and cardiac autonomic function. In: Stansfeld SA, Marmot MG, editors. Stress and the Heart: psychosocial pathways to coronary heart disease. London, UK: BMJ Books; 2002. p. 221–55. 10.1053/euhj.2000.2534 11428849

[13] Ussher JM. Women’s Health: Contemporary International Perspectives. 1st ed. Leicester, UK: The British Psychological Society; 2000.

[14] Steptoe A, Demakakos P, de Oliveira C, Wardle J. Distinctive biological correlates of positive psychological well-being in older men and women. Psychosom Med. 2012 Jun;74(5):501–8. doi: 10.1097/PSY.0b013e31824f82c8 22511728

[15] Shah AS V, Griffiths M, Lee KK, McAllister DA, Hunter AL, Ferry A V, et al. High sensitivity cardiac troponin and the under-diagnosis of myocardial infarction in women: prospective cohort study. BMJ. 2015 Jan 21;g7873. doi: 10.1136/bmj.g7873 25609052 PMC4301191

[16] Karppinen H, Laakkonen ML, Strandberg TE, Tilvis RS, Pitkälä KH. Will-to-live and survival in a 10-year follow-up among older people. Age Ageing. 2012 Nov;41(6):789–94. doi: 10.1093/ageing/afs082 22762904

[17] Kiecolt-Glaser JK, McGuire L, Robles TF, Glaser R. Emotions, morbidity, and mortality: new perspectives from psychoneuroimmunology. Annu Rev Psychol. 2002;53:83–107. doi: 10.1146/annurev.psych.53.100901.135217 11752480

[18] Lee KH, Xu H, Wu B. Gender differences in quality of life among community-dwelling older adults in low- and middle-income countries: results from the Study on global AGEing and adult health (SAGE). BMC Public Health. 2020 Dec 28;20(1):114. doi: 10.1186/s12889-020-8212-0 31992264 PMC6988191

[19] Brazier J, Jones N, Kind P. Testing the validity of the Euroqol and comparing it with the SF-36 health survey questionnaire. Quality of Life Research. 1993;2(3). doi: 10.1007/BF00435221 8401453

[20] Ware JE, Sherbourne CD. The MOS 36-item short-form health survey (SF-36). I. Conceptual framework and item selection. Med Care. 1992 Jun;30(6):473–83. 1593914

[21] Skevington SM, Sartorius N, Amir M. Developing methods for assessing quality of life in different cultural settings. Soc Psychiatry Psychiatr Epidemiol. 2004 Jan 1;39(1):1–8. 15022040 10.1007/s00127-004-0700-5

[22] Reeve BB, Wyrwich KW, Wu AW, Velikova G, Terwee CB, Snyder CF, et al. ISOQOL recommends minimum standards for patient-reported outcome measures used in patient-centered outcomes and comparative effectiveness research. Quality of Life Research. 2013 Oct 4;22(8):1889–905. doi: 10.1007/s11136-012-0344-y 23288613

[23] Diener E, Lucas RE. Explaining differences in societal levels of happiness: Relative standards, need fulfilment, culture and evaluation theory. J Happiness Stud. 2000;1(1):41–78.

[24] Skevington SM, Böhnke JR. How is subjective well-being related to quality of life? Do we need two concepts and both measures? Soc Sci Med. 2018 Jun;206:22–30. doi: 10.1016/j.socscimed.2018.04.005 29680769

[25] Cummins RA. Personal Income and Subjective Well-being: A Review. J Happiness Stud. 2000;1(2):133–58.

[26] Carstensen LL. A Life-Span Approach to Social Motivation. In: Motivation and Self-Regulation across the Life Span. Cambridge University Press; 1998. p. 341–64.

[27] Helliwell J, Putnam RD. The social context of well-being. In: Huppert FA, Baylis N, Keverne B, editors. The Science of Well-Being. Oxford, UK: Oxford University Press; 2005. p. 435–60.

[28] Ryff CD, Singer B. Middle age and well-being. In: Friedman HS, editor. Encyclopedia of mental health. New York: Academic Press; 1998. p. 707–19.

[29] Huppert FA. A Population Approach to Positive Psychology: The Potential for Population Interventions to Promote Well-Being and Prevent Disorder. In: Linley PA, Joseph S, editors. Positive Psychology in Practice. Chichester, UK: John Wiley; 2004. p. 693–712.

[30] Office for National Statistics (ONS). ONS website, statistical bulletin. 2022 [cited 2023 Aug 30]. Personal well-being in the UK: April 2021 to March 2022. Available from: https://www.ons.gov.uk/peoplepopulationandcommunity/wellbeing/bulletins/personalwellbeingintheukquarterly/april2011toseptember2021 (30 August 2023)

[31] Skevington SM, Lotfy M, O’Connell KA, WHOQOL Group. The World Health Organization’s WHOQOL-BREF quality of life assessment: psychometric properties and results of the international field trial. A report from the WHOQOL group. Qual Life Res. 2004 Mar;13(2):299–310. doi: 10.1023/B:QURE.0000018486.91360.00 15085902

[32] The WHOQOL Group. The World Health Organization Quality of Life assessment (WHOQOL): position paper from the World Health Organization. Soc Sci Med. 1995 Nov;41(10):1403–9. doi: 10.1016/0277-9536(95)00112-k 8560308

[33] The WHOQOL Group. Development of the World Health Organization WHOQOL-BREF quality of life assessment. Psychol Med. 1998 May;28(3):551–8. 9626712 10.1017/s0033291798006667

[34] Skevington SM O’Connell KA, WHOQOL Group. Can we identify the poorest quality of life? Assessing the importance of quality of life using the WHOQOL-100. Qual Life Res. 2004 Feb;13(1):23–34. doi: 10.1023/B:QURE.0000015317.71791.be 15058784

[35] Molzahn AE, Kalfoss M, Schick Makaroff K, Skevington SM. Comparing the importance of different aspects of quality of life to older adults across diverse cultures. Age Ageing. 2011 Mar 1;40(2):192–9. doi: 10.1093/ageing/afq156 21186234

[36] Skevington SM, Epton T. How will the sustainable development goals deliver changes in well-being? A systematic review and meta-analysis to investigate whether WHOQOL-BREF scores respond to change. BMJ Glob Health. 2018;3. doi: 10.1136/bmjgh-2017-000609 29379649 PMC5759710

[37] Feder K, Michaud DS, Keith SE, Voicescu SA, Marro L, Than J, et al. An assessment of quality of life using the WHOQOL-BREF among participants living in the vicinity of wind turbines. Environ Res. 2015;142. doi: 10.1016/j.envres.2015.06.043 26176420

[38] Ballew M, Leiserowitz A, Maibach E, Marlon J. Yale Program on Climate Change Communication. 2018. Gender Differences in Public Understanding of Climate Change. Available from: https://climatecommunication.yale.edu/publications/gender-differences-in-public-understanding-of-climate-change/

[39] The WHOQOL Group. The development of the World Health Organisation quality of life assessment instrument (The WHOQOL). In: Orley J, Kuyken W, editors. Quality of Life Assessment: International Perspectives. Berlin: Springer-Verlag; 1994. p. 41–60.

[40] Ratner PA, Sawatzky R. Approaches to the measurement of gender. In: Oliffe JL, Greaves L, editors. Designing and Conducting Gender, Sex, and Health Research. 2011.

[41] The Whoqol Group. The World Health Organization quality of life assessment (WHOQOL): Development and general psychometric properties. Soc Sci Med. 1998 Jun;46(12):1569–85. doi: 10.1016/s0277-9536(98)00009-4 9672396

[42] WHOQOL SRPB Group. A cross-cultural study of spirituality, religion, and personal beliefs as components of quality of life. Soc Sci Med. 2006 Mar;62(6):1486–97. doi: 10.1016/j.socscimed.2005.08.001 16168541

[43] WHOQOL-OLD Group, Power M, Quinn K, Schmidt S. Development of the WHOQOL-old module. Qual Life Res. 2005 Dec;14(10):2197–214. doi: 10.1007/s11136-005-7380-9 16328900

[44] WHOQOL HIV Group. WHOQOL-HIV for quality of life assessment among people living with HIV and AIDS: results from the field test. AIDS Care. 2004 Oct;16(7):882–9. doi: 10.1080/09540120412331290194 15385243

[45] Skevington SM. Advancing cross-cultural research on quality of life: observations drawn from the WHOQOL development. World Health Organisation Quality of Life Assessment. Qual Life Res. 2002 Mar;11(2):135–44. 12018737 10.1023/a:1015013312456

[46] IBM. SPSS. 2018.

[47] Bowden A, Fox-Rushby JA. A systematic and critical review of the process of translation and adaptation of generic health-related quality of life measures in Africa, Asia, Eastern Europe, the Middle East, South America. Soc Sci Med. 2003 Oct;57(7):1289–306. doi: 10.1016/s0277-9536(02)00503-8 12899911

[48] Skevington S. M., et al. (2021). "Enhancing the multi-dimensional assessment of quality of life: introducing the WHOQOL-Combi." Qual Life Res 30(3): 891–903. doi: 10.1007/s11136-020-02661-9 33331967 PMC7952286

[49] Skevington SM. Qualities of life, educational level and human development: an international investigation of health. Soc Psychiatry Psychiatr Epidemiol. 2010 Oct;45(10):999–1009. doi: 10.1007/s00127-009-0138-x 19820887

[50] Nussbaum MC. Women and Human Development. Cambridge, UK: Cambridge University Press; 2000.

[51] World Health Organization (WHO). Women and Health: today’s evidence, tomorrow’s agenda [Internet]. Geneva: World Health Organization; 2009 [cited 2023 Oct 20]. Available from: https://iris.who.int/handle/10665/44168

[52] UNESCO. Online programme and meeting document UNESCO’s Gender Mainstreaming Implementation Framework (GMIF) for 2002–2007 [Internet]. Section for Women and Gender Equality B of SP, editor. Paris: UNESCO; 2003. Available from: https://unesdoc.unesco.org/ark:/48223/pf0000131854

[53] UNESCO. Handbook for gender focal points in UNESCO National Commissions [Internet]. Section for Women and Gender Equality B of SP, editor. Paris: UNESCO; 2005. Available from: https://unesdoc.unesco.org/ark:/48223/pf0000140572

[54] Gupta GR, Oomman N, Grown C, Conn K, Hawkes S, Shawar YR, et al. Gender equality and gender norms: framing the opportunities for health. Vol. 393, The Lancet. 2019. doi: 10.1016/S0140-6736(19)30651-8 31155276

[55] Jaime-Lara RB, Koons BC, Matura LA, Hodgson NA, Riegel B. A Qualitative Metasynthesis of the Experience of Fatigue Across Five Chronic Conditions. J Pain Symptom Manage. 2020 Jun;59(6):1320–43. doi: 10.1016/j.jpainsymman.2019.12.358 31866485 PMC7239763

[56] Taylor S. E., et al. (2000). "Biobehavioral responses to stress in females: tend-and-befriend, not fight-or-flight." Psychol Rev 107(3): 411–429 doi: 10.1037/0033-295x.107.3.411 10941275

[57] Umberson D, Karas Montez J. Social Relationships and Health: A Flashpoint for Health Policy. J Health Soc Behav. 2010 Mar 8;51(1_suppl):S54–66. doi: 10.1177/0022146510383501 20943583 PMC3150158

[58] Curtis S. Health and Inequality: Geographical Perspectives. London, United Kingdom: SAGE Publications, Ltd; 2004.

[59] Gall A, Anderson K, Howard K, Diaz A, King A, Willing E, et al. Wellbeing of Indigenous Peoples in Canada, Aotearoa (New Zealand) and the United States: A Systematic Review. Int J Environ Res Public Health. 2021 May 28;18(11). doi: 10.3390/ijerph18115832 34071636 PMC8198891

[60] Sala OE, Meyerson LA, Parmesan C, editors. Biodiversity change and human health: from ecosystem services to spread of disease. Washington DC: Island Press; 2009.

[61] Michie S, van Stralen MM, West R. The behaviour change wheel: A new method for characterising and designing behaviour change interventions. Implementation Science. 2011 Dec 23;6(1):42. doi: 10.1186/1748-5908-6-42 21513547 PMC3096582

[62] O’Connell KA, Skevington SM. Spiritual, religious, and personal beliefs are important and distinctive to assessing quality of life in health: a comparison of theoretical models. Br J Health Psychol. 2010 Nov;15(Pt 4):729–48. doi: 10.1348/135910709X479799 19948086

[63] Pargament KI. The psychology of religion and coping: Theory, research, practice. Guildford, UK: Guildford Press; 1997.

[64] Magar V. (2015). "Gender, health and the Sustainable Development Goals." Bull World Health Organ 93(11): 743. doi: 10.2471/BLT.15.165027 26549898 PMC4622165

